# Investigating nitrous oxide leaks at St George Hospital: A case study using the discrepancy and pressure testing methods

**DOI:** 10.1177/0310057X251379095

**Published:** 2025-12-10

**Authors:** Rebecca Cregan, Kristen Pickles, Philomena Colagiuri, Scott McAlister, Forbes McGain, Katy Bell

**Affiliations:** 1 NSW Ministry of Health Climate Risk and Net Zero Unit, Sydney, Australia; 2 Wiser Healthcare Research Collaboration, The University of Sydney, Sydney, Australia; 3 The Healthcare Carbon Lab, University of Melbourne, Parkville, Australia; 4 Departments of Anaesthesia and Intensive Care, Western Health, Victoria, Australia; 5 Department of Critical Care, University of Melbourne, Victoria, Australia

**Keywords:** Nitrous oxide, anaesthetic gases, occupational exposure, carbon footprint, global warming, analgesia, healthcare

## Abstract

Nitrous oxide (N_2_O) is ozone-depleting and a greenhouse gas. Studies have shown a high wastage of N_2_O from leaking hospital infrastructure. Identifying leaks is a priority action in the Australian national health and climate strategy. Four possible methods to identify leaks have been described: the discrepancy method, cylinder weighing, pressure testing, and flow monitoring. We used the discrepancy and pressure testing methods to investigate possible N_2_O leaks at St George Hospital, a large tertiary hospital in Sydney providing medical, surgical, birthing, paediatric, and trauma care. Our investigation was in four steps: (a) to determine how much N_2_O is procured and calculate the associated carbon emissions; (b) to outline the location of pipeline supply; (c) to determine how much N_2_O is used clinically (operating theatres, delivery suite, other areas); and (d) to assess for leaks throughout the pipeline using pressure testing. We estimated a total annual ‘worst case’ estimate of maximum possible clinical N_2_O use of approximately 801,866 litres at St George Hospital in 2021, with 14,846 litres used in the operating theatres and 787,020 litres used in the delivery suite. This estimate was approximately 319,534 litres (or 28%) less than the 1,121,400 litres procured N_2_O used to supply the manifold, indicating leaks at least this large. Pressure testing of the full manifold system identified leaks in three operating theatres. A substantial amount of the N_2_O procured by St George Hospital is leaking to the atmosphere causing unnecessary emissions. This N_2_O provides no benefits to clinical care, has financial costs, and may pose potential occupational exposure risks to clinicians.

## Introduction

Nitrous oxide (N_2_O) is a gas used clinically as a common adjunct to analgesia in labour, as well as for procedural sedation, and decreasingly, as an optional component of general anaesthesia.^1–3^ It is also a greenhouse gas, with a global warming potential 274 times greater than carbon dioxide, and an atmospheric lifetime of approximately 100 years.^4,5^ With the successful phase out of chlorofluorocarbons, N_2_O is now also the dominant ozone-depleting substance released into the atmosphere. Occupational exposure among clinical staff working in areas where the gas is used for patient care may increase the risk of adverse health outcomes. Occupational exposure has been associated with an increased risk of spontaneous abortion and adverse neonatal outcomes (exposure during pregnancy), adverse neurological and psychiatric effects, and other adverse outcomes.^6^ Although scavenging systems are routinely used in Australian hospitals to mitigate these risks, they do not address leaks in the pipeline prior to the point of the anaesthetic machine and are infrequently available in the delivery suite to address leaks from N_2_O/oxygen blenders.

For medical use, N_2_O is a schedule 4 (prescription only) medicine, meaning that its possession, prescription, and supply is limited to certain health practitioners and authorised persons only. Nitrous oxide also poses the potential for recreational abuse, which necessitates careful storage and security practices to prevent theft. In 2022, Australia’s therapeutic goods administration re-classified N_2_O as a schedule 6 poison for non-therapeutic use. This means that manufacturers must now follow packaging and labelling rules and apply mandatory warnings, and the purchaser must be aged 16 years or older. The use of N_2_O as a recreational drug may be increasing, as well as harms related to this use.^7^

In the UK, emissions associated with anaesthetic gases account for 2–5% of the total healthcare footprint, with the majority due to N_2_O.^8^ Recently published Australian data indicate substantial carbon dioxide equivalent (CO_2_e) emissions due to purchased N_2_O, with large variations across states and territories that is unlikely to be attributable to differences in clinical use.^9^ Efforts in Scotland to reduce emissions from N_2_O revealed that waste from piped manifold systems is often a far larger problem than clinical usage; for example, leaking infrastructure accounted for 80% of procured N_2_O (685,000 litres per annum, equivalent to 359 tonnes CO_2_e) in one hospital.^10^ Leaks occur at the manifold connection itself, or at outlets downstream from the manifold, especially if an outlet valve is in situ or within the piped infrastructure. Leaks in manifold systems have been identified in many parts of the world,^4,11,12^ including Australia.^13^ These leaks cause large amounts of N_2_O to be released into the internal hospital environment and then the atmosphere, with an associated financial cost and zero health benefit.

The elimination of N_2_O leaks is a priority action for governments to achieve the Australian government-legislated net zero plan targets for healthcare (National Health and Climate Strategy, Action 4.13).^14^

Because N_2_O is colourless and not pungent, leaks need to be actively detected, and the Interim Australian Centre for Disease Control (CDC) has proposed four methods to do so. These are the discrepancy method, cylinder weighing, pressure testing, and flow monitoring.^15^ This study aimed to use two of these methods—the discrepancy method and pressure testing—to investigate possible N_2_O leaks at St George Hospital, a 627-bed major tertiary teaching hospital in Sydney, New South Wales (NSW), providing medical, surgical, birthing, paediatric, and trauma care. This case study may assist clinicians and management teams to identify, minimise, and prevent N_2_O leaks in their own facilities to reduce environmental impacts, financial loss, and prevent potential occupational exposure. The project was led by a NSW health net zero clinical lead (RC) with academic support from the University of Sydney health services researchers (KB, PC, SM, KP) as part of the Wiser Healthcare Net Zero partnership.^16^

## Methods

In the Australian state of NSW, N_2_O is supplied commercially in cylinders of varying sizes (935 litres to 18,690 litres) which then provide gas either directly at the point of patient care, or through a central manifold system which is accessed via wall outlets. Large cylinders are connected to a pipeline network that originates in a central location and branches to provide pipeline supply to clinical areas where N_2_O is used. Additionally, N_2_O is provided in some hospital locations as entonox (fixed gas mixture of 50% oxygen and 50% N_2_O; this is not provided at St George Hospital). Specifications for installation, commissioning, and maintenance of N_2_O infrastructure are covered under Australian standards. In NSW, all maintenance and works on these pipeline systems are performed by the external gas supplier (or subsidiary).

This study was granted exemption from ethics approval by the South Eastern Sydney Local Health District Human Research Ethics Committee (23 May 2023).

We used four steps in our investigation of possible N_2_O leaks at St George Hospital. These were: (a) to determine how much N_2_O is procured for the facility and calculate the associated carbon emissions; (b) to outline the location of pipeline supply; (c) to determine how much N_2_O is being used clinically (operating theatres, delivery suite, other areas); and (d) to assess for leaks throughout the pipeline. (These steps are presented in detail in Supplementary file 1).

## Results

To determine how much N_2_O is procured for the facility and calculate the associated carbon emissions.

In the calendar year 2021, St George Hospital procured a total of 1,177,885 litres (2235 kg) of N_2_O, as shown in Table 1.^17^ This represents a carbon footprint of 610 tonnes CO_2_e.

**Table 1.** Procured nitrous oxide at St George Hospital in 2021.

Cylinder type^a^	Volume of N_2_O (litres)	Number purchased	Total (litres)
C	935	19	17,765
D	3520	11	38,720
G	18,690	60 (10 × 6-pack)	1,121,400

a C cylinders are small, portable cylinders; D cylinders are large portable cylinders; G cylinders are extra-large cylinders available only as a pack of six and supply the manifold pipeline.^17^

### Outline the location of pipeline supply

**Figure 1.**
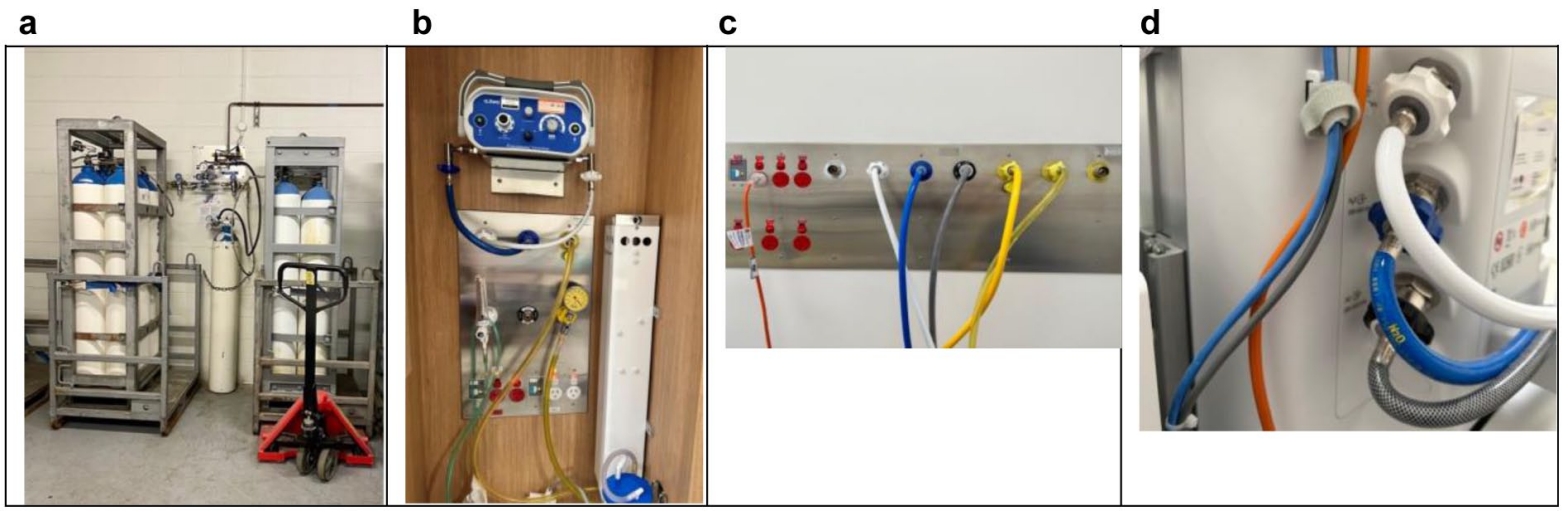
Piped nitrous oxide supply for clinical use at St George Hospital, Sydney, New South Wales. (a) Central nitrous oxide manifold; (b) Delivery suite nitrous oxide blender with adjacent scavenging system; (c) Sample gas wall outlet; (d) Piped gas connection to anaesthetic ventilator.

There are strict regulatory requirements for the storage of N_2_O cylinders, given the potential for supply interruption and abuse (AS2896:2021).^18^ At St George Hospital, the central manifold and the storage area for N_2_O cylinders are located in a large, locked, well-ventilated area close to the hospital loading dock. The manifold is supplied by packs of six G-sized cylinders (18,690 litres N_2_O per cylinder). There are two packs of cylinders connected to the system, as illustrated in Figure 1. One pack provides pipeline supply. Once the pressure in the ‘in-use’ pack falls below 650 kPa, the system will automatically rotate to the alternative pack and alarm to change the emptied pack.

The N_2_O pipeline supply splits from the main pipeline to supply three hospital buildings with current or historical N_2_O use (the latter being areas with no current clinical use and an old valve system). At St George Hospital, the areas supplied with current clinical use are the operating theatres in two buildings, delivery suite, emergency department, cardiac catheter laboratory, radiology, and the Cancer Care Centre. One area with historical use had a pipeline supply: anatomical pathology. The modern clinical areas have comprehensive computer aided draft schemas for the pipeline supply, indicating the location of the pipelines, valves and pressure gauges, making potential sites for leak easy to locate and remedy if necessary. The hospital’s supplier of piped N_2_O reports deficiencies in the whole pipeline mapping, which includes legacy supply in older buildings, and this deficiency in mapping is likely to exist in older parts of all hospitals. There are older sections of pipeline which are susceptible to leak but not easy (and potentially not possible) to locate. Where there have been works performed, and changes in building structure and purpose that have occurred before good record keeping, there are likely to be inconsistencies and incomplete pipeline maps. Emergency department N_2_O use was not included in the calculations as our initial consultations with representatives indicated that it is used infrequently in the emergency department at St George Hospital (see Supplementary file 1, step 3, other areas).

### Determine how much nitrous oxide is being used for patient care

#### Operating theatres

##### Survey of anaesthetists practising at St George Hospital

A staff survey (see Supplementary file 2) was conducted from 17 July 2023 to 1 August 2023. Of the 129 anaesthetists we invited to complete the online survey, 63 completed responses were returned (49% response rate). Of those who completed the survey, 79% (50 of 63) indicated that they had used N_2_O as part of a general anaesthetic or to supplement sedation in the last 12 months (at any hospital that they worked at), and 71% (45 of 63) reported that their frequency of use had not changed in the last 5 years. Of those indicating that their frequency of use had decreased, many reported that this occurred once they had become aware of the adverse environmental impacts of N_2_O (see Supplementary file 3).

Of those who reported using N_2_O, 36% (18 of 50) used N_2_O less than one to two times per month, 44% (22 of 50) used it one to two times per month, 14% (seven of 50) used it one to two times per week, and 6% (three of 50) used N_2_O one to two times per day. Nitrous oxide was most used for paediatric patients (84%, 42 of 50), followed by obstetric patients (36%, 18 of 50), elderly patients (36%, 18 of 50), ‘sick’ patients (26%, 13 of 50), other (24%, 12 of 50), and complex patients (12%, six of 50) (respondents were able to answer multiple categories, so percentages sum to more than 100%). Free text responses indicated that where N_2_O was used for paediatric cases, this was for induction and then switched off (Supplementary file 3). Several respondents also indicated that the N_2_O use that they reported was for other hospitals they worked at, not St George Hospital, suggesting the survey data were likely to be an overestimate of clinical use of N_2_O at the hospital.

To estimate the proportion of all cases in the operating theatres at St George Hospital where N_2_O was used, we calculated a denominator for the survey data in which anaesthetists were asked about N_2_O use across all of the hospitals they worked at. We calculated this as the total cases for 63 anaesthetists across all hospitals that they worked, per week: 63 respondents each provided anaesthesia for approximately 30 cases per week, which equals 1890 total cases per week. Using the upper limits of N_2_O use frequency options in the survey, we calculated the numerator as: 13 respondents × 0 cases per week + 18 respondents × 0.25 cases per week + 22 respondents × 0.5 cases per week + 7 respondents × 2 cases per week + 3 respondents × 10 cases per week = 59.5 cases per week. This results in an estimated proportion of 3% of all cases in operating theatres using N_2_O (N_2_O used in 59.5 cases of 1890 cases per week; likely to be a ‘worst’ case estimate representing maximum possible clinical use).

##### Caseload determination

The Surginet database (an electronic patient record system) indicated that the total number of cases conducted in operating theatres in 2021 was 16,225. Case numbers are split into adult and paediatric numbers in Table 2.

**Table 2.** Number of operations performed at St George Hospital in 2021.

Specialty	Number of cases
Paediatric surgery	78
Adult surgery (including obstetrics)	16,147
Total	16,225

##### Estimation of clinical use of N_2_O in operating theatres

Multiplying total cases by 3% (see above), results in approximately 487 cases in operating theatres that used N_2_O in 2021. Multiplying 487 cases by 30.5 litres N_2_O per case (mean volume of N_2_O used, when it is used in a case)^2^ results in approximately 14,846 litres of N_2_O used in operating theatres in 2021 for anaesthesia.

#### Delivery suite

##### Number of births

There were 2498 births at St George Hospital in 2021. Of these, there were 623 that were spontaneous with no augmentation, 1440 with augmented labour (all forms), and 435 with no labour (elective Caesarean sections) (Table 3). Augmentation of labour is noteworthy in this context as it frequently increases analgesic requirement directly, as well as indirectly through increased risk of intervention. There were also 384 emergency Caesarean sections.

**Table 3.** Nitrous oxide used during labour and delivery.

Nitrous oxide use	Number of parturients	% of total births
Any nitrous oxide used	1149	46%
Nitrous oxide only	258	10.3%
Nitrous oxide + epidural (± other pain management)	538	21.5%
Nitrous oxide + other (no epidural)	353	14%

Fourteen per cent of patients utilised both N_2_O and other non-epidural forms of analgesia, such as local anaesthetic and opioids. This group also included patients having spinal anaesthesia with supplementary N_2_O and may include patients who were in labour prior to Caesarean section and patients who had elective Caesarean sections but required additional analgesia.

##### Calculation of underestimation of N_2_O use in the eMaternity birth summary data

A random sample of 100 birth summary records in the eMaternity database for deliveries recorded as not using N_2_O was manually cross-checked against labour and delivery electronic progress notes in the Surginet database. This found that of 100 women who apparently did not use N_2_O according to the eMaternity data, 24 patients did actually use N_2_O during labour, but this was not recorded in the birth summary data. The other 76 women were correctly recorded as not using N_2_O. To account for this under-reporting in the eMaternity data, we revised the estimated proportion of deliveries without N_2_O use (54%) downwards by a correction factor of 0.76 (76 of 100) to arrive at an estimate of 41% of deliveries that were without N_2_O use. Subtracting this from 100% resulted in an estimated 59% of deliveries in which N_2_O was used.

##### Estimation of clinical use of N_2_O in delivery suite

Multiplying the total 2498 deliveries (see above) by 59% (see above) results in approximately 1474 deliveries in which N_2_O was used in 2021. Multiplying this by 534 litres per delivery (mean volume of N_2_O used, when it is used in a delivery)^18^ results in approximately 787,020 litres of N_2_O used in the delivery suite in 2021.

##### Total clinical use (operating theatres plus delivery suite)

Operating theatre ‘worst case’ use was estimated to be 14,846 litres of N_2_O and delivery suite ‘worst case’ use was estimated to be 787,020 litres (‘worst case’ represents the maximum possible clinical use). This resulted in a total annual ‘worst case’ estimate of clinical N_2_O use of approximately 801,866 litres at St George Hospital in 2021. This is the hypothetical maximum possible use, and we expect that actual use is likely to be lower than this. This estimate is approximately 319,534 litres (or 28%) less than the 1,121,400 litres of procured N_2_O in G cylinders used to supply the manifold, indicating that leaks in the manifold system were responsible for at least this volume of N_2_O.

### Assess for leaks throughout the pipeline

All pipelines throughout the hospital were pressure tested after the pipelines were isolated from the cylinder supply. Change in pressure was detected via measurements on pressure gauges throughout the system before and after isolation. Leaks were identified in three operating theatres by a drop in pressure recorded over 4 hours via individual gas pressure monitors for each theatre. There was a pressure drop of approximately 40 kPa, 55 kPa and 25 kPa over 4 hours in the affected theatres. The volume of this leak over time could not be calculated as we did not have measurements of the length of the pipeline, nor a flow meter to measure ongoing loss. Temperatures were not measured during testing in these areas but were assumed not to have changed significantly over the 4 hours of testing, so the pressure drop is likely to be attributable to leaking infrastructure. No leaks were identified for the remainder of the pipeline system, including all other operating theatres and individual clinical rooms in the delivery suite (noting that possible leaks in the oxygen/N_2_O blenders in the delivery suite would not be detected by pressure testing).

## Discussion

In 2021, St George Hospital procured a total of 1,177,885 litres of N_2_O, with a carbon footprint of 610 tonnes CO_2_e. Most of this was used to supply piped N_2_O in the manifold (1,121,400 litres procured N_2_O in G cylinders). Our ‘worst case’ maximum possible clinical use estimates accounted for approximately 801,86 litres of N_2_O. Using the discrepancy method, this indicates that leaks in the manifold system wasted at least 28% of the procured N_2_O. This indicates leaks of at least 319,534 litres or 171 tonnes CO_2_e per year, equivalent to driving a standard medium petrol car 432,911 km, or driving around Australia ten times (https://ecoinvent.org/ecoinvent-v3-10/). We found that most of the clinical use was for maternity services (‘worst case’ maximum estimate 787,020 litres (98% of N_2_O used clinically), with a regular but relatively infrequent use of N_2_O as an adjunct to general anaesthesia in operating theatres at St George Hospital (‘worst case’ maximum estimate 14,846 litres (2% of N_2_O used clinically)). Using the direct pressure testing method, we identified leaks in the manifold system in three operating theatres. The precise location of these leaks within the theatres is still under investigation.

Nitrous oxide leaks are difficult to measure, and accurate and current N_2_O clinical use data are challenging to obtain in Australia. We attempted to overcome these obstacles using two methods to identify N_2_O leaks, including in a high-use obstetric setting, providing a case example so that others may use these methods in their own settings. The identified leak represents a substantial amount of greenhouse gas emissions that could be avoided without any impact on clinical care.

To our knowledge, this is the first detailed report of a hospital-wide pressure test as a method to detect N_2_O leaks. Through a systematic search of the literature, we found published examples of the other three methods proposed by the interim Australian Centre for Disease Control, 2024^15^—discrepancy, cylinder weighing, and flow monitoring (search strategy presented in Supplementary file 4). Three studies used the discrepancy method as we did,^11,19,20^ one study used the cylinder weighing method,^21^ and one study used flow monitoring.^21^ These studies, and their relevance to the current study, are described below.

The first of the three studies using the discrepancy method is a recent audit in a quaternary care hospital in Saudi Arabia^11^ estimated clinical N_2_O use by extracting data from electronic anaesthetic records rather than relying on self-assessment as in our study (electronic data on N_2_O use were not available to us). These authors found a very large discrepancy (more than 98%) between procurement (3,950,000 litres per year) and clinical use (72,871 litres per year) leading to a recommendation to decommission the medical gas pipeline system for N_2_O and replace the supply with portable cylinders. The second study is an Australian study in a non-obstetric, non-paediatric hospital in Melbourne, Victoria, estimated a ‘highest use of N_2_O scenario' by interrogating ‘super user’ data (available to approved users, commonly biomedical engineers) from an anaesthetic machine and extrapolating to the department for a 3-year period.^20^ They found a nearly 80% discrepancy between procurement (208,000 litres per year) and clinical use (43,440 litres per year). The authors then used a liquid detergent gas detector to identify a faulty O-ring on a wall outlet that accounted for at least part of the leak.

The third study using the discrepancy method is another Australian study in a different hospital in Melbourne. The authors estimated the clinical use of N_2_O in operating theatres, the birthing suite, and paediatric emergency department using clinical audit, and modified sleeves of a portable flow meter.^19^ They found a 21% discrepancy between procurement (86,600 litres per month) and clinical use (147,000 litres per month), and like us, they found the highest use was by maternity services (95% of clinical use). The authors conducted leak testing (liquid detergent testing—see below) and localised (non-hospital wide) pressure testing, but the authors were not able to locate an obvious leak during preliminary investigations.

The study that used the cylinder weighing method to identify leaks was conducted in a Melbourne tertiary non-obstetric hospital. Authors compared clinical use information (from electronic anaesthesia records and flow meters on clinical anaesthetic machines) with changes in N_2_O cylinder weights (recorded 12-hourly).^21^ The researchers identified a very large N_2_O leak (83.5%). The study that used the flow monitoring method was conducted in a tertiary paediatric-only hospital in NSW. The authors installed a continuous flow meter connected between a N_2_O cylinder pack and the hospital manifold.^22^ Leaks of approximately half a litre per minute were identified during periods of no clinical activity; overall this was equivalent to an approximately 50% leak of the procured N_2_O.

Current manifold testing recommended by the Australian standard does not appear to be adequate to identify leaks that develop in pipelines after they have been commissioned. The manifold is inspected every 6 months and valves are ‘snoop tested’ with liquid detergent and soapy water (which theoretically bubbles if a leak is present) every 2 years. As is seen in our case study and other examples described above, this can often fail to detect leaks. Some hospitals are investigating replacing existing pipelines with discrete, smaller cylinders used at the point of patient care. This has recently been completed at the Prince Charles Hospital in Brisbane (a tertiary non-obstetric centre with a minimal paediatric case mix).^23^ Decommissioning piped N_2_O manifold systems is likely to be the most effective way to decrease N_2_O wastage.^2,9,12^ A recent report from the USA of 47 US hospitals in an academic (UCSF-Health) and a private (Providence) health system, found that leaks accounted for 47.2% to 99.8% of purchased N_2_O.^12^ These results supported 25 of the 47 hospitals to transition to portable N_2_O supply systems with deactivation of central supply, including hospitals with paediatric and obstetric services. There were no clinical, operational, or logistical issues noted because of the transitions. However, in the USA, it is rare to supply N_2_O centrally for labour and delivery analgesia services, with 46 of the 47 hospitals using portable supplies for these services even before decommissioning of the central supply systems. In Australia, achieving agreement to decommission piped N_2_O systems may be more difficult for hospitals with significant obstetric and paediatric services than for hospitals not providing these services. There are also potential storage and security issues associated with relying solely on cylinders which will need to be addressed. Experience in the UK has identified higher than expected cylinder turnover following the removal of its piped N_2_O systems, likely to be secondary to theft and inadequate staff training in medical gas cylinder management.^24^

While there has been much published discussion in the anaesthetic literature about the pattern of (and declining) N_2_O use, there has not been an equivalent volume of publication and discussion in obstetric and maternity literature. Nitrous oxide is used in most vaginal deliveries, sometimes over many hours and in significant volumes. Using a portable flow meter, Wong et al. (2022) calculated an average of 534 litres of N_2_O used per labour and estimated clinical use in obstetrics to be 95% of their hospital N_2_O use.^19^ We estimated it to account for 98% of clinical use in the current study. When used as an adjunct to analgesia in vaginal delivery, N_2_O increases the carbon footprint of the delivery by 25 times.^25^ There is an urgent need for research to find acceptable alternatives to N_2_O for use in labour, identify the barriers and facilitators to moving to these alternatives, and evaluating interventions that are co-designed with women who are preparing for, or have recently given birth, and the clinicians who provide their maternity care.

The strengths of our study include a multidisciplinary collaboration with acadmics, clinicians and non-clinical staff at the hospital, which has led to greater shared awareness not only of the potential for leaks in the manifold but also awareness of the environmental harms associated with N_2_O. Multiple sources of evidence were utilised to assess clinical uses. Although there were several challenges associated with pressure testing the manifold (see below), this has added useful information for developing practical solutions to evaluating pipelines and identifying leaks. This study also provides valuable data on N_2_O emissions from anaesthesia practices in Australia, of which there are few other published estimates.^2^ Although there were significant pandemic-related measures during the study period (2021), including the suspension of elective surgery in the second half of that year, this was an advantage for application of the discrepancy method. As leaks accounted for a larger proportion of purchased N_2_O in 2021 (less clinical use than in other years,^26^ but similar procurement),^9^ the size of the leak was easier to quantify.

There are also several limitations to our study. We were unable to interrogate N_2_O use data from anaesthetic machines which would have provided objective data on the clinical use of N_2_O, and instead had to rely on self-reporting by a sample of anaesthetists practising at the hospital. As they also worked at multiple other hospitals, and our survey was designed to be short to maximise our response rate, there is considerable uncertainty in the actual clinical use estimates. Practice may differ across different institutions according to case mix and other local factors. In addition, the survey was conducted in 2023, but the purchasing data, surgical case data, and birthing data were all for 2021. This means that survey data are not specific to the timeframe of the procurement and clinical use data, where providers may have been using different amounts of N_2_O (the survey showed that nearly 30% of respondents had changed their use of N_2_O in the past 5 years). As most of the clinical use was in the delivery suite, and we used birthing data from the same year as the procurement data (2021), it is unlikely that the timing of the survey would have a large effect on the estimated discrepancy between the amount purchased and used. We also did not account for residual N_2_O in manifold cylinders that are returned to suppliers in our leak estimates, although these volumes are likely to have been small.^12^ Finally, we made several assumptions to calculate the clinical use of N_2_O, including mean volume estimates for N_2_O use from other published studies. For N_2_O used as an adjunct to general anaesthesia in adults, there were no Australian data available and our estimate (30.5 litres per case if N_2_O was used) is from data for the National Health Service England in 2017 (see Table 1 in Liu et al.).^2^ Those data are for use in adults, and there are no empirical data available for use in paediatric anaesthesia; however, modelling suggests that amounts used per case are the same or lower than in adults.^2,27^ For N_2_O used for labour analgesia, our estimate (534 litres per labour) is from data in an Australian study conducted at Western Health, Melbourne, in 2020–2021.^19^

There were significant logistical challenges to coordinating testing which involved an engineering workforce with a heavy workload through routine operations as well as hospital redevelopment, hospital gas suppliers with similar workload concerns, and the need to coordinate the appropriate safety disruption notices with other necessary hospital works.

There is an industry-wide shortage of licensed medical gas technicians in NSW who were needed to provide testing oversight, while the staff from the engineering department who carry out the testing were occupied with their routine engineering workload.

A limitation of both the pressure testing and discrepancy methods is that they are unable to detect the precise location of leaks (the cylinder weighing method is also unable to locate the leak, only the flow monitoring method can). Mapping the pipeline found deficiencies in the historical record of infrastructure. While the leaks identified in our pressure test were in actively used and accurately mapped segments of the pipeline, there are likely to be additional smaller leaks that were not detected, including in anaesthetic gas blenders such as those used in the delivery suite. Given the difficulties in establishing a testing protocol and conducting the pressure testing to identify pipeline leaks, this may not be the most efficient way to establish the existence of a leak in the pipeline system. There were also several capital works projects in progress at the hospital during the testing period which presented further challenges to the study.

## Conclusion

At least 28% of the piped N_2_O that is purchased at St George Hospital is leaked, representing a substantial amount of carbon emissions that could be avoided without any impact on clinical care. Leaks were identified in three operating theatres.

## Supplemental Material

sj-docx-1-aic-10.1177_0310057X251379095 – Supplemental material for Investigating nitrous oxide leaks at St George Hospital: A case study using the discrepancy and pressure testing methods Supplemental material, sj-docx-1-aic-10.1177_0310057X251379095 for Investigating nitrous oxide leaks at St George Hospital: A case study using the discrepancy and pressure testing methods by Rebecca Cregan, Kristen Pickles, Philomena Colagiuri, Scott McAlister, Forbes McGain and Katy Bell in Anaesthesia and Intensive Care
