# Supplementary material for: Investigating nitrous oxide leaks at St George Hospital: A case study using the discrepancy and pressure testing methods
Source: Anaesth Intensive Care. 2025 Dec 10;54(3):235–42. doi: 10.1177/0310057X251379095 (PMC13161490; doi:10.1177/0310057X251379095)
Supplement: sj-docx-1-aic-10.1177_0310057X251379095 – Supplemental material for Investigating nitrous oxide leaks at St George Hospital: A case study using the discrepancy and pressure testing methods [file sj-docx-1-aic-10.1177_0310057X251379095.docx]

# Supplementary files

## Supplementary 1: Detailed description of 4 steps used to detect nitrous oxide leaks at St George Hospital, Sydney

**Step 1: Determine how much nitrous oxide is procured for the facility and calculate the associated carbon emissions**

Nitrous oxide procurement data was obtained through the NSW Ministry of Health Climate Risk & Net Zero Unit. The calendar year 2021 was chosen for the study period. Pandemic related measures in 2021, including the suspension of elective surgery in the second half of that year, mean that any potential leaks would account for a larger proportion of total nitrous purchased by the hospital, making the size of the leak easier to quantify. The greenhouse gas emissions from for the total nitrous oxide purchased for the year was calculated by converting total volume of nitrous oxide in litres in procured cylinders to kilograms. The 2021 Intergovernmental Panel on Climate Change (IPCC) conversion factor (273 kg CO2e/kg) was then used to convert this to equivalent kilograms of carbon dioxide (CO_2_e) [1]. All procured nitrous is included in this footprint calculation as any residual nitrous in cylinders returned to the supplier is vented to atmosphere prior to refilling.

**Step 2: Outline location of pipeline supply**

Hoslab Medical & Laboratory Gas Systems [2], the nitrous oxide supplier for St George Hospital, was engaged to provide pipeline infrastructure information, including maps of existing cylinder storage facilities, manifolds, pipelines, valves, and wall outlets. Understanding where the infrastructure is located is important as leaks are frequently located at any site there is an interruption to the copper pipeline, such as valves, joins, ceiling pendants and outlets. Leaks have also been identified in flexible hosing (made of braided nylon with PVC coating) connecting the outlets and pendants to anaesthetic ventilators [3].

**Step 3: Determine how much nitrous oxide is being used for patient care**

Operating Theatres:

1. Survey of anaesthetists practising at St George Hospital

An online survey (Supplementary 3) was developed by the study team and distributed to clinicians in the anaesthetic department at St George Hospital, including consultants and trainees. External trainees such as residents and ICU/ED registrars who are less familiar with anaesthetic practice were not invited to complete the survey. Invitations were distributed via a link directly e-mailed to individual anaesthetists and QR codes were displayed throughout the anaesthetic department for two weeks. To maximise responses, we designed the survey to be short (4 questions) and easy to respond to. The intent was to estimate clinical use of nitrous oxide through self-reported usual practice. We did not directly ask about environmental concerns, although respondents could voice these concerns in the free text response question if they wanted to.

1. Caseload determination

A search of the SurgiNet database – an electronic record of all perioperative or procedural care cases done within Operating Theatres - was conducted to identify (a) total number of operations for 2021, (b) duration of procedure, and (c) subspecialty type to complement the anaesthetist survey.

To estimate the clinical use of nitrous oxide in operating theatres, we used data from the clinician survey to estimate the percentage of theatre cases where nitrous oxide is used. We estimated ‘worst case’ scenarios taking the maximum possible estimates of clinical use using the survey data. We then multiplied this number by 30.5L (estimated to be the mean volume of nitrous used, when it is used in a case [4], to arrive at the approximate volume used for cases in operating theatres.

Delivery suite:

Data for number of deliveries for 2021 was obtained from the Mothers and Babies Report 2021 [5]. We also searched de-identified records from eMaternity of summary birth data for St George hospital in 2021, to identify deliveries where nitrous oxide use was recorded. eMaternity is the electronic perinatal data collection system used by St George Public Hospital to record “demographic, medical and obstetric information on the mother and the condition of the infant” (HealthStats NSW Perinatal Data Collection). We noted the type of delivery: vaginal delivery (with or without induction of labour /augmentation) or caesarean section (with and without labour). Induction of labour in this context refers to the mechanical or pharmacological process of initiating uterine contractions to commence labour. This may include balloons or prostaglandins applied to the cervix, rupture of membranes and oxytocin infusions. Augmentation of labour is the process of artificially stimulating the uterus to increase the strength and/or frequency of contractions to help progress labour (e.g. through rupture of membranes and oxytocin infusions).

Following conversations between the PI (RC) and delivery suite staff, we hypothesised that some nitrous use may not have been captured in the eMaternity summary data, which would have impacted our assessment of clinical use (leading to underestimation). To evaluate this, a random sample of 100 birth summary records from eMaternity that did not have nitrous oxide use documented were manually cross checked (by medical record number) against labour and delivery electronic progress notes in Surginet to determine how often nitrous oxide was used but not recorded in summary documentation. We assigned all 2021 eMaternity records a random number, ordered records without nitrous use recorded numerically by assigned random number, and selected the first 100 records to review. We used the findings from these 100 cases to estimate the proportion of births with no apparent nitrous use according to eMaternity data, but where there was some nitrous use according to Surginet case notes. We used this to adjust the eMaternity results for nitrous use in 2021 upwards, to more accurately capture the number of births using nitrous. We estimated ‘worst case’ scenarios taking the maximum possible estimates of nitrous oxide use for obstretic cases.

We then multiplied the number of births using nitrous by 534L, the estimate for nitrous use per birth reported in a study by Wong et. Al [6]. These authors used a flow meter in their delivery suite nitrous pipeline to directly measure nitrous volumes.

Other areas:

To capture clinical use of piped nitrous for procedures that were not captured in the Surginet database of operating theatre cases, the PI (RC) held discussions with representatives from the Emergency Department and the Paediatric Department about the frequency of nitrous oxide use in these departments (discussions included paediatric trauma cases). Nitrous was reported to be infrequently used in both of these areas. When it was used, this was from the piped supply in ED but via cylinder at point of care for Paediatrics. While there is piped nitrous oxide available for use in general anaesthesia in the cardiac catheter labs, cancer care centre and radiology, these represent a small number (<5%) of the total cases performed and are unlikely to contribute significantly to the total nitrous oxide use in anaesthesia. The ventilator used for paediatric MRI sessions utilises a portable nitrous oxide cylinder when required as there is no accessible wall outlet for nitrous oxide in the MRI suite (i.e. no piped nitrous). Oral health is not located on campus at St George hospital, and so was not included.

**Step 4: Assess for leaks throughout the pipeline**

Pressure testing of the entire nitrous oxide piped manifold system by isolation over 4 hours is considered the gold standard for gas leak assessment. The process is in line with commissioning requirements for the pipeline defined by the Australian Standard AS2896:2021. This testing can detect leaks in the whole hospital manifold system. It does not identify the precise location of the leaks. The adaptation of this methodology was established in collaboration with Hoslab, the nitrous oxide supplier for St George Hospital.

The PI (RC) held multiple consultative meetings with relevant stakeholder groups, including anaesthetics, maternity, engineering, hospital operations and Hoslab to establish the testing protocol. We considered whether to test only the whole manifold or to conduct additional testing and isolate different buildings and sections of the pipeline, and in what timeframe (one big testing window or multiple smaller tests with less impacted users). Conducting testing on smaller sections of pipeline was proposed to identify any leak identified on the whole system test. An option to terminate the supply to redundant sections of pipeline by blocking them off prior to testing and reduce potential sites for leaks was considered, but excluded based on timing, funding and simplicity.

Saturday morning was chosen to undertake the testing, to minimise disruption to maternity services. The date chosen was coordinated with availability of required licenced medical gas technicians and the existing schedule of hospital disruption notices associated with both routine maintenance and ongoing capital works on site. Affected groups including operating theatres and delivery suite were provided with written information prior to testing, as well as support personnel on location during the testing. Approval sign-off was obtained from hospital executive and relevant heads of department. The project had additional executive sponsorship from the South Eastern Sydney Local Health District Environmental Sustainability Steering Committee.

On the day of testing, and prior to the isolation of the pipeline system from the manifold, oxygen-nitrous oxide blenders in delivery suite were disconnected from the reticulation system to exclude possible issues with the blenders contributing to any potential leaks identified. Portable nitrous oxide cylinders with pressure regulators were provided to delivery suite, operating theatres, and the emergency department. Staff were reminded that there was a possibility of a low-pressure alarm in the nitrous oxide system only, and any additional gas alarms were not part of the testing and should be acknowledged in the usual manner.

Isolation valves in the pipeline to supplied areas of the hospital were recorded in a table. Areas without pressure gauges at the valve box had these fitted at the terminal gas outlet (generally a wall outlet) in the affected area of supply (see Supplementary 2). Pressures were recorded throughout the pipeline system before the pipeline was isolated from the cylinder supply, as well as immediately following isolation and at the conclusion of the 4-hour test. Pressure gauges had tape applied where the needle rested in the analogue display at the start of the test and were photographed to be compared at the conclusion. At the conclusion of the testing, all affected areas were notified that the pipeline system had returned to usual operating conditions.

## Supplementary 2: Staff survey

| Question | Response options |
| --- | --- |
| In your clinical practice, have you used nitrous oxide as part of a general anaesthetic or supplement to sedation in the last 12 months? | - Yes - No |
| How frequently would you use nitrous oxide? | - 1-2 times per day - 1-2 times per week - 1-2 times per month - Less frequently - Please specify |
| In which patients would you typically use nitrous oxide as part of your anaesthetic? | - Paediatrics - Obstetrics - Elderly patients - Sick patients - Complex pain patients - Other (please specify) |
| Have you changed the frequency that you use nitrous oxide in the past 5 years? | - Yes - No |
| Please tell us what prompted the change, and any other information about nitrous that you would like to tell us. | Free text |

## Supplementary 3: Free text survey responses

| **How frequently would you use N2O (please specify) (n=13)** | Inhalation induction children - usually off for maintenance  1-2 times per year for paediatric inhalational induction  GA LSCS   Some paeds cases  1-3 times a year  Part of a inhalational induction for the odd child that turns up on my list. I switch to air for maintenance though  Adjunct to analgesia for cesarean regional anaesthesia and paediatrics inductions routinely  Only for gas inductions in children - and I have done it only once in the last 12 months and not at St George  Probably once every 6 months  Paediatric inductions. Rarely have this case  Occasional vascular patient who needs more inhalational agent but also has a bad heart/ vasculature etc so nitrous helps increase MAC without increasing the agent very occasionally use it to demonstrate the effect on MAC in a healthy patient (up to 10 mins during a case)  Very rare use at St George Public. Might use for short periods to augment analgesia for short painful procedure.  Also use for gas induction for paediatrics - though rare for me at St George public  Use in paeds (rarely at St G) but more frequently at other centres. Extremely rare use in obstetrics - prior to conversion to GA for LSCS with failing block (either at maternal request or offered as option)  Only use it for the occasional paediatric induction. Otherwise don't use it much at all. |
| --- | --- |
| **‘Other’ types of patients for which clinician typically uses N2O as part of their anaesthetic (n=12)** | Some v anxious day stay patients  As part of inhalational induction in adults as well as children  I use it for high turnover gynae and dental lists on LMAs, cant get pt deep enough/quick enough to not move for instrumentation - (and also keep them spont breathing!) without it  Trauma surgery involving haemodynamic instability.  I ticked Obstetrics for GA C sections only.  Healthy painful cases ie bum abscess  Gas induction eg no or difficult IV access  Sedation for Ivc  Showing registrars how it is used  Orthopaedics, High risk PONV. Gynae  Special Needs Adult Dental Patients (not at St George Public Hospital)  Short spontaneously breathing but intensely painful procedures in young men. Bum absess  Very short high stimulating procedure - perianal abscess. Once the bum cut done, nitrous oxide turned off. Patient still wake relatively quickly. |
| **What prompted change in frequency of N2O use in the past 5 years and any other info about N2O that you would like to tell us (n=17)** | The environment  Started to do a regular paeds list  Environment  More concerned re climate effects of nitrous oxide than the PONV effects - always felt this was a little over-emphasised.  Increased utilisation  I stopped using it regularly in 2010 because I found there are other adjuncts to maintain a decent MAC without all the side effects of Nitrous Oxide  Reduced use of nitrous in last 5 years. Dont do kids list or ortho trauma anymore can't remember when I last used it  Environmental  Effect on environment, equally efficacious alternatives for most Anaesthesia and analgesia  I just don't think there's any real reason to use any more.  More judicious and considered use for patients who have a clear indication or clear benefit from using nitrous oxide.  Use less due to it's detrimental environmental effects  Damn greenies making me feel guilty about it. Seriously though, I think it's important to consider carefully it's use and be responsible about when to add it in  Now use as frequently but in lower volumes with ultra-low flow anaesthesia  Environmental impact of N2O use has led me to using less  It makes you sick and heats the planet  not really needed (perhaps only in maternity) -ve environmental impact |

## Supplementary 4: Search strategy for published applications of methods for detecting nitrous oxide leaks in hospitals

Our search strategy was designed and developed with the help of a health librarian at the University of Sydney. We searched databases MEDLINE via Ovid, Embase via Ovid, CINAHL via Ebsco and Scopus from inception to 7th May 2024. There were no restrictions on language or time of publication.

**Scopus**

hospital* OR clinical OR "operat* room*" OR "operat* theat*" OR anesth* OR anaesth* OR surg* AND "nitrous Oxide" AND (leak* OR fumes OR exposure OR waste OR "pressure test*") AND ((health OR adverse) W/2 (impact* OR effects OR affect OR symptom*)) OR "occupational safety" OR sustainab* OR environment* OR "net zero" OR emission* OR footprint OR "greenhouse gases" AND guideline* OR framework* OR strategy OR strategies OR policy OR policies OR toolkit* OR procedure* OR protocol* OR monitor* OR assess* OR evaluat* OR procur*

**Embase via Ovid**

hospital* OR clinical OR "operat* room*" OR "operat* theat*" OR anesth* OR anaesth* OR surg* AND "nitrous Oxide" AND (leak* OR fumes OR exposure OR waste OR "pressure test*") AND ((health OR adverse) W/2 (impact* OR effects OR affect OR symptom*)) OR "occupational safety" OR sustainab* OR environment* OR "net zero" OR emission* OR footprint OR "greenhouse gases" AND guideline* OR framework* OR strategy OR strategies OR policy OR policies OR toolkit* OR procedure* OR protocol* OR monitor* OR assess* OR evaluat* OR procur*

**CINAHL via Ebsco**

hospital* OR clinical OR "operat* room*" OR "operat* theat*" OR anesth* OR anaesth* OR surg* AND "nitrous Oxide" AND (leak* OR fumes OR exposure OR waste OR "pressure test*") AND ((health OR adverse) W/2 (impact* OR effects OR affect OR symptom*)) OR "occupational safety" OR sustainab* OR environment* OR "net zero" OR emission* OR footprint OR "greenhouse gases" AND guideline* OR framework* OR strategy OR strategies OR policy OR policies OR toolkit* OR procedure* OR protocol* OR monitor* OR assess* OR evaluat* OR procur*

**MEDLINE via Ovid**

hospital* OR clinical OR "operat* room*" OR "operat* theat*" OR anesth* OR anaesth* OR surg* AND "nitrous Oxide" AND (leak* OR fumes OR exposure OR waste OR "pressure test*") AND ((health OR adverse) W/2 (impact* OR effects OR affect OR symptom*)) OR "occupational safety" OR sustainab* OR environment* OR "net zero" OR emission* OR footprint OR "greenhouse gases" AND guideline* OR framework* OR strategy OR strategies OR policy OR policies OR toolkit* OR procedure* OR protocol* OR monitor* OR assess* OR evaluat* OR procur*

## References

1. Masson-Delmotte, V., et al., *Climate change 2021: the physical science basis.* Contribution of working group I to the sixth assessment report of the intergovernmental panel on climate change, 2021. **2**(1): p. 2391.

2. *Hoslab Pty Ltd*. Available from: <https://hoslab.com.au/>.

3. Jain, A., et al., *An unusual defect in the nitrous oxide pipeline.* Journal of Anaesthesiology Clinical Pharmacology, 2012. **28**(2): p. 267-268.

4. Liu, Y., et al., *Nitrous oxide use in Australian health care: strategies to reduce the climate impact.* Anesthesia & Analgesia, 2023. **137**(4): p. 819-829.

5. *Centre for Epidemiology and Evidence. New South Wales Mothers and Babies 2021*. 14 June 2023.

6. Wong, A., et al., *Quantitative nitrous oxide usage by different specialties and current patterns of use in a single hospital.* British Journal of Anaesthesia, 2022. **129**(3): p. e59-e60.

7. Peters, M.D.J., et al., *Updated methodological guidance for the conduct of scoping reviews.* JBI Evidence Synthesis, 2020. **18**(10): p. 2119-2126.

8. Chakera, A., *Driving down embedded emissions from medical nitrous oxide*. 2021, British Medical Journal Publishing Group.

9. Gaff, S.J., V.X. Chen, and E. Kayak, *A weighing method for measuring nitrous oxide leakage from hospital manifold-pipeline networks.* Anaesthesia and Intensive Care, 2024. **52**(2): p. 127-130.

10. Kayak, E., et al., *Detecting and reducing nitrous oxide leaks in healthcare facilities: a practical guide*. 2024, Interim Australian Centre for Disease Control, Department of Health and Aged ….

11. Skowno, J.J., et al., *Hospital‐level flow measurement to detect nitrous oxide leakage.* Anaesthesia, 2024.

12. Majeed, A. and A.M. Awan, *Should pipeline nitrous oxide be discontinued in secondary care: A cost-benefit analysis.* Saudi Journal of Anaesthesia, 2024. **18**(2): p. 194-196.

13. Seglenieks, R., et al., *Discrepancy between procurement and clinical use of nitrous oxide: waste not, want not.* British Journal of Anaesthesia, 2022. **128**(1): p. e32-e34.
